# Factors influencing post-traumatic stress disorder among police officers in South Korea

**DOI:** 10.3389/fpubh.2022.1024284

**Published:** 2022-12-09

**Authors:** Hye-Kyung Oh, Cheol Yeung Jang, Mi Suk Ko

**Affiliations:** ^1^Department of Nursing, Daegu University, Daegu, South Korea; ^2^Department of Police Administration, Daekyeung University, Gyeongsan, South Korea; ^3^Department of Social Welfare, Corea Welfare Cyber University, Gyeongsan, South Korea

**Keywords:** post-traumatic stress disorder, Korean police officers, critical incident trauma, social support, resilience

## Abstract

**Objectives:**

This study aims to identify critical incident trauma (CIT), social support, resilience, and post-traumatic stress disorder (PTSD) in Korean police officers and to determine factors related to PTSD to obtain basic data for developing a PTSD intervention.

**Methods:**

A mixed-methods approach was adopted by administering structured questionnaires to Korean police officers and conducting semi-structured interviews with seven Korean police stakeholders. The structured questionnaires elicited information on CIT, social support, resilience, and PTSD. Data from 189 participants were analyzed using independent *t*-tests, Pearson's correlation coefficients, and multiple linear regression analysis. The interview data, which elicited information on difficulties and coping strategies after CIT, police organizational culture, current status of counseling programs, and suggestions for PTSD interventions, were analyzed using an inductive thematic analysis.

**Results:**

Factors that are significantly related to PTSD (28.7% of variance) are very healthy subjective health status (B = −0.44, *p* = 0.013), CIT (B = 0.18, *p* ≤ 0.001), social support (B = −0.38, *p* ≤ 0.001), and resilience (B = −0.18, *p* = 0.044). The stakeholders revealed the following PTSD-related factors: the difficulties and limitations of overcoming traumatic experiences, coping strategies, police counseling program status, and opinions on PTSD-related programs.

**Conclusion:**

Perceived health status, CIT, social support, and resilience had a strong relationship with PTSD. Therefore, the success of PTSD interventions for Korean police officers should be considered.

## Introduction

Police service is a risky and stressful occupation that involves frequent exposure to confrontation and violence, which are potentially harmful and can be a source of traumatic experiences for police personnel. Exposure to traumatic experiences can lead to various mental health complications, including post-traumatic stress disorder (PTSD) (1). Trauma may occur as a result of life-threatening events, such as car accidents, interpersonal violence, and manmade and natural disasters (2). Police officers are more often exposed to these situations than other occupational groups.

In a traumatic incident, a critical incident trauma (CIT) is any traumatic situation in which the service personnel experience unusually strong emotional reactions that interfere with their ability to exert control over the situation. It occurs when police officers face a crisis in the work environment that produces either immediate or delayed stress (3). Previous studies revealed that CIT plays an important role in the development of PTSD in police officers (1, 4). Currently, the suicide rate of police officers is approximately 20 deaths per 100,000 persons, which is approximately three times the suicide rate of public officials in South Korea (5, 6). Police officers face many challenges that affect their mental health, which may affect the important role they play in public policing; therefore, it is important to identify factors that influence police officers' PTSD.

One of the factors that may influence PTSD among police officers is social support. Social support is a multidimensional construct and refers to the various resources provided through interpersonal relationships (7). Even in high-risk situations, if appropriate social support is obtained, PTSD can be prevented and reduced among veterans and military service members (8). Among police officers, a lack of social support increases a sense of isolation and has been found to be correlated with a higher level of stress (3) and PTSD (9–11).

Similar to social support, resilience is a complex construct that encapsulates the process and outcomes of successful adaptation to difficult or adverse life events (12, 13). Resilience is a significant predictor of PTSD symptoms among paramedics (14). As for police officers, some foreign studies have shown that resilience significantly decreased PTSD symptoms (2, 10).

Several studies have identified the current status of PTSD and systemic coping strategies (9, 15), the relationships between CIT and PTSD (16), and resilience and PTSD (17) in police officers. However, in South Korea, few studies have been conducted on the factors affecting PTSD among police officers. In this study, we identified the factors that influence PTSD among police officers using questionnaires and interviews to develop a PTSD intervention for police officers in South Korea.

## Materials and methods

### Study design

This study adopted a mixed-methods approach to identify the influencing factors of PTSD in police officers in South Korea. The quantitative design involved administering structured questionnaires to police officers, while the qualitative design involved conducting semi-structured video interviews with seven stakeholders (five police officers, one clinical psychologist, and one professor of police administration).

### Participants

The participants were police officers who expressed a desire to voluntarily participate in the study. They were recruited using convenience sampling. Questionnaire surveys were conducted online from May to June 2022. For the quantitative part, the sample size was calculated using G^*^power 3.1.9.4, with a significant level of 0.05, medium effect size of 0.15, power of 0.95, and 13 independent variables. The minimum sample size was 189. After completing the questionnaire, the final sample size was 189. For the qualitative part, three nursing professors and two police officers prepared a list of relevant stakeholders through meetings. Semi-structured interviews were conducted from May to July 2022 using a video conferencing program.

This study was conducted after obtaining approval from the institutional review board (Approval no: 1040621-202111-HR-066) of Daegu University in Korea. We explained the purpose, methods, and procedure of the study; the confidentiality and anonymity of the data; and the possibility of withdrawing participation at any time for any reason. Written informed consent to participate in the study was obtained online.

### Variables and instruments

Ten items on participants' characteristics (sex, age, education level, religion, affiliated working department, workplace, rank, work, field experience, and subjective health status) were constructed by the researchers based on previous studies. The Life Event Checklist for Diagnostic and Statistical Manual of Mental Disorder-5 (DSM-5) by the American National Center for PTSD was used to assess participants' CIT (18). The checklist assesses exposure to 17 events known to potentially result in PTSD or distress; however, we excluded 2 inappropriate questions such as those related to combat or exposure to a war zone in the military or as a civilian and severe human suffering. These ambiguous items were excluded because our study's participants were not military soldiers. The scoring was also modified from the existing 1–6 scale to a 1–5 scale (ranging from “doesn't apply” to “happened to me”). Because the survey was completed by police officers, the scale “Part of my job” was excluded. Social support was measured using the Korean version of the Interpersonal Support Evaluation list-12 (ISEL-12) (19). This scale comprises 12 questions, with each question rated on a 4-point Likert scale (ranging from “absolutely not true” to “absolutely true”). Resilience was measured using the resilience research center's adult resilience measure (RRC-ARM) developed by Liebenberg and Moore (20). It consists of eight elements (social/community inclusion, family attachment and support, spirituality, national and cultural identity, and personal competencies) and 28 items. Each question has a 5-point Likert scale ranging from 1 (“not at all”) to 5 (“extremely”). PTSD was assessed using the Korean version of the PTSD checklist-5 (PCL-5-K) developed by Kim et al. (21). It is a 20-item self-report measure that assesses the presence and severity of PTSD symptoms and corresponds with the Diagnostic and Statistical Manual of Mental Disorder-5 (DSM-5) criteria for PTSD. Severity was determined by adding the scores of each item to determine a total score (ranging from 0 to 80). A total score of 33 or higher suggests that the participants needed further assessment to confirm their diagnosis of PTSD. Each question uses a Likert scale ranging from 0 (“not at all”) to 4 (“extremely”).

### Statistical analyses

All collected data were analyzed using SPSS Statistics 22.0 (IBM Corp., Armonk, MY, USA). We described participants' demographic characteristics, CIT, social support, resilience, and PTSD using real numbers, percentages, means, and standard deviations. Cronbach's α coefficient was used to verify the internal reliability of the measures. We analyzed the differences in the participants' CIT, social support, resilience, and PTSD according to their general characteristics using independent *t*-tests. Correlations among CIT, social support, resilience, and PTSD were analyzed using Pearson's correlation coefficients. Finally, factors that influence participants' PTSD were analyzed using multiple linear regression analyses. The dependent variable in these analyses was PTSD, and the independent variables were general characteristics, CIT, social support, and resilience. The interview data were analyzed using an inductive thematic analysis approach for thematic content analysis.

## Results

### General characteristics

Table 1 presents the general characteristics of the 189 police officers who participated in this study. The majority are male (80.4%) and aged between 30 and 39 years (37.6%). Of the participants, 60.9% had completed a university degree, 19.0% had completed high school, 10.6% had completed a postgraduate degree, and 9.5% had completed a college degree. Approximately two-thirds of the participants are atheists. Additionally, approximately 21.7% of the police officers worked in the police precinct or police box, while 16.9, 11.1, 7.4, 7.4, 6.9, 6.3, 5.8, 4.8, 4.2, 2.7, and 2.1% worked in the investigation, detective, intelligence, women and juvenile affairs, police administration, community safety, public security, traffic division, emergency dispatching and operations command center, mobile police, and audit and inspection departments, respectively. As for rank, approximately half of the participants were assistant inspectors (22.2%) or inspectors (30.7%). Regarding work experience, the highest proportion (27.5%) had 5 to < 10 years of experience, while the smallest proportion (4.8%) had 25 to < 30 years of experience. As for the field experience of participants, the highest proportion (37.5%) had < 5 years, while the smallest proportion (5.3%) had over 25 years. Regarding subjective health status, the majority of participants perceived themselves to be generally healthy.

**Table 1:** Demographic characteristics of subjects (*N* = 189).

**Characteristics**	**Classification**	**Frequency (%)**
Sex	Male	152 (80.4)
	Female	37 (19.6)
Age (years)	20–29	25 (13.2)
	30–39	71 (37.6)
	40–49	57 (30.2)
	50 and over	36 (19.0)
Education level	High school	36 (19.0)
	College	18 (9.5)
	University	115 (60.9)
	Postgraduate or above	20 (10.6)
Religion	Christianity	16 (8.5)
	Catholicism	19 (10.0)
	Buddhism	40 (21.2)
	None	114 (60.3)
Affiliated working department	Police administration division	13 (6.9)
	Community safety division	12 (6.3)
	Women and juvenile affairs division	14 (7.4)
	Investigation division	32 (16.9)
	Detective division	21 (11.1)
	Public security division	11 (5.8)
	Traffic division	9 (4.8)
	Intelligence division	14 (7.4)
	Emergency dispatching and operations command center	8 (4.2)
	Police precinct or police box	41 (21.7)
	Audit and inspection department	4 (2.1)
	Mobile police	5 (2.7)
	Etc.	5 (2.7)
Rank	Policeman	24 (12.7)
	Senior policeman	32 (16.9)
	Assistant Inspector	42 (22.2)
	Inspector	58 (30.7)
	Senior Inspector	28 (14.8)
	Above superintendent	5 (2.7)
Work experience	< 5 years	32 (16.9)
	5 < 10 years	52 (27.5)
	10 < 15 years	32 (16.9)
	15 < 20 years	32 (16.9)
	20 < 25 years	20 (10.6)
	25 < 30 years	9 (4.8)
	Over 30 years	12 (6.4)
Field experience	< 5 years	71 (37.5)
	5 < 10 years	57 (30.2)
	10 < 15 years	20 (10.6)
	15 < 20 years	18 (9.5)
	20 < 25 years	13 (6.9)
	Over 25 years	10 (5.3)
Subjective health status	Having severe or some disease	21 (11.1)
	Normal	43 (22.8)
	Generally healthy	93 (49.2)
	Very healthy	32 (16.9)

### CIT, social support, resilience, and PTSD

The mean scores with corresponding SDs were 2.34 ± 1.02 (out of 5), 2.97 ± 0.49 (out of 4), 3.58 ± 0.56 (out of 5), and 1.05 ± 0.65 (out of 4) for CIT, social support, resilience score, and PTSD, respectively (Table 2).

**Table 2:** Critical incident trauma, social support, resilience, and post-traumatic stress disorder.

**Variable**	**M ±SD**	**Min**	**Max**	**Cronbach's α**
Critical incident trauma	2.34 ± 1.02	1	5	0.932
Social support	2.97 ± 0.49	1	4	0.890
Resilience	3.58 ± 0.56	1	5	0.955
Post-traumatic stress disorder	1.05 ± 0.65	0	4	0.960

SD, standard deviation.

### Differences in CIT, social support, resilience, and PTSD according to general characteristics

As shown in Table 3, we found significant differences in CIT scores based on sex (t = 5.12, *p* = 0.025), work experience (F = 2.20, *p* = 0.045), field experience (F = 2.66, *p* = 0.024), and subjective health status (F = 2.86, *p* = 0.038). An ex-post analysis revealed no significant differences between the groups. We also found differences in social support based on age (F = 4.28, *p* = 0.006), rank (F = 3.06, *p* = 0.011), work experience (F = 2.54, *p* = 0.022), and subjective health status (F = 12.24, *p* ≤ 0.001). The results of an ex-post analysis revealed that participants aged 20 years had higher social support scores than those aged 50 years and over. Among the rank levels, the policeman group (the lowest rank in South Korea) had higher social support scores than the senior inspector group. Regarding subjective health status, participants with a very healthy status showed significantly higher social support scores than those with other health statuses. Participants with a normal health status had significantly lower social support scores than those with a generally healthy status.

**Table 3:** Critical incident trauma, social support, resilience, and post-traumatic stress disorder according to general characteristics.

**Characteristics**	**Critical incident trauma**			**Social support**			**Resilience**			**Post-traumatic stress disorder**		
	**M ±SD**	**t/F**	***p***	**M ±SD**	**t/F**	***p***	**M ±SD**	**t/F**	***p***	**M ±SD**	**t/F**	***p***
**Sex**												
Male	2.42 ± 1.01	5.12	0.025	2.96 ± 0.49	0.35	0.553	3.57 ± 0.54	0.13	0.716	1.05 ± 0.63	0.10	0.758
Female	2.00 ± 0.98			3.01 ± 0.52			3.61 ± 0.62			1.02 ± 0.74		
Age												
20–29^a^	2.05 ± 0.95	2.24	0.085	3.23 ± 0.51	4.28	0.006	3.65 ± 0.59	1.44	0.232	0.76 ± 0.67	4.23	0.006
30–39^b^	2.36 ± 0.99			2.97 ± 0.48		a > d^‡^	3.47 ± 0.58			0.97 ± 0.70		a < d^‡^
40-49^c^	2.23 ± 1.03			2.96 ± 0.50			3.65 ± 0.49			1.11 ± 0.61		
50 and over^d^	2.67 ± 1.02			2.78 ± 0.44			3.65 ± 0.58			1.31 ± 0.53		
**Education level**												
High school completed^a^	2.43 ± 0.88	0.36	0.785	2.99 ± 0.45	0.54	0.655	3.57 ± 0.44	3.38	0.020	1.04 ± 0.57	0.27	0.848
College completed^b^	2.30 ± 0.94			3.06 ± 0.57			3.44 ± 0.61		c < d^‡^	1.03 ± 0.58		
University completed^c^	2.29 ± 1.08			2.96 ± 0.51			3.55 ± 0.59			1.03 ± 0.71		
Postgraduate or above^d^	2.50 ± 1.00			2.86 ± 0.42			3.94 ± 0.38			1.17 ± 0.56		
**Rank**												
Policeman^a^	2.40 ± 1.03	0.37	0.868	3.21 ± 0.53	3.06	0.011	3.69 ± 0.51	0.67	0.644	0.74 ± 0.70	2.29	0.047
Senior policeman^b^	2.24 ± 1.01			2.98 ± 0.54		a > e^‡^	3.55 ± 0.52			1.08 ± 0.79		
Assistant inspector^c^	2.33 ± 1.02			3.07 ± 0.39			3.55 ± 0.64			0.94 ± 0.62		
Inspector^d^	2.29 ± 1.04			2.89 ± 0.50			3.55 ± 0.52			1.14 ± 0.57		
Senior inspector^e^	2.55 ± 1.00			2.75 ± 0.43			3.56 ± 0.58			1.27 ± 0.62		
Above superintendent^f^	2.19 ± 1.12			2.97 ± 0.59			3.94 ± 0.48			0.89 ± 0.56		
**Work experience**												
< 5 years	2.28 ± 0.96	2.20	0.045	3.21 ± 0.50	2.54	0.022	3.63 ± 0.50	0.74	0.621	0.74 ± 0.68	2.43	0.027
5 < 10 years	2.24 ± 1.05			2.96 ± 0.48			3.51 ± 0.59			0.97 ± 0.68		
10 < 15 years	2.48 ± 1.03			3.00 ± 0.42			3.64 ± 0.54			1.07 ± 0.67		
15 < 20 years	2.17 ± 0.94			2.94 ± 0.58			3.50 ± 0.57			1.19 ± 0.62		
20 < 25 years	2.13 ± 1.05			2.78 ± 0.39			3.54 ± 0.52			1.17 ± 0.63		
25 < 30 years	2.48 ± 1.08			2.84 ± 0.65			3.71 ± 0.60			1.44 ± 0.29		
Over 30 years	3.25 ± 0.78			2.72 ± 0.34			3.80 ± 0.58			1.23 ± 0.49		
**Field experience**												
< 5 years^a^	2.06 ± 0.95	2.66	0.024	3.03 ± 0.52	1.84	0.108	3.53 ± 0.60	0.62	0.687	0.85 ± 0.67	3.38	0.006
5 < 10 years^b^	2.35 ± 1.03			2.99 ± 0.50			3.56 ± 0.54			1.05 ± 0.67		a < d^‡^
10 < 15 years^c^	2.49 ± 1.04			3.05 ± 0.41			3.77 ± 0.44			1.13 ± 0.67		
15 < 20 years^d^	2.65 ± 0.96			2.87 ± 0.40			3.62 ± 0.41			1.44 ± 0.49		
20 < 25 years^e^	2.65 ± 0.96			2.69 ± 0.40			3.57 ± 0.67			1.11 ± 0.55		
Over 25 years^f^	3.00 ± 1.02			2.73 ± 0.56			3.64 ± 0.69			1.41 ± 0.32		
**Subjective health status**												
Having severe or some disease^a^	2.83 ± 0.89	2.86	0.038	2.72 ± 0.38	12.24	< 0.001 a, b, c < d, b < c^‡^	3.56 ± 0.49	6.56	< 0.001 b < c, d^‡^	1.45 ± 0.49	9.79	< 0.001 a,b,c > d, a > c^‡^
Normal^b^	2.50 ± 1.15			2.74 ± 0.44			3.28 ± 0.58			1.21 ± 0.65		
Generally healthy^c^	2.22 ± 0.97			3.00 ± 0.47			3.66 ± 0.51			1.02 ± 0.62		
Very healthy^d^	2.14 ± 0.95			3.32 ± 0.47			3.78 ± 0.57			0.61 ± 0.62		

‡ Scheffe.

a − f means the sub-categories of each variable.

Resilience scores differed significantly across education levels (F = 3.38, *p* = 0.020) and subjective health status (F = 6.56, *p* < 0.001). An ex-post analysis revealed that, for education level, participants with a university degree had higher resilience scores than those with a postgraduate degree. In terms of subjective health status, participants with normal health status had lower resilience scores than those in the general and very healthy status groups. We also found differences in PTSD according to age (F = 4.23, *p* = 0.006), rank (F = 2.29, *p* = 0.047), work experience (F = 2.43, *p* = 0.027), field experience (F = 3.38, *p* = 0.006), and subjective health status (F = 9.79, *p* ≤ 0.001). The results also revealed that participants aged 20–29 years had lower PTSD scores than those aged 50 years and over. As for field experience, participants who had <5 years of experience had lower PTSD scores than those who had 15 to <20 years of experience. For subjective health status, participants who had a very healthy status had lower PTSD scores than those who had other health statuses. Participants who had severe or some diseases had higher PTSD scores than those who were generally healthy.

### Relationship between CIT, social support, resilience, and PTSD

The correlations between the major variables are listed in Table 4. CIT had a significant positive correlation with resilience (r = 0.19, *p* = 0.009) and PTSD (r = 0.30, *p* #x02264; 0.001). Social support showed a significant positive correlation with resilience (r = 0.48, *p* < 0.001) and a significant negative correlation with PTSD (r = −0.40, *p* < 0.001). Resilience had a negative correlation with PTSD (r = −0.23, *p* < 0.001).

**Table 4:** Correlations between critical incident trauma, social support, resilience, and post-traumatic stress disorder.

**Variable**	**1**	**2**	**3**	**4**
1. Critical incident trauma	1			
2. Social support	0.03 (0.669)	1		
3. Resilience	0.19 (0.009)	0.48 (<0.001)	1	
4. Post–traumatic stress disorder	0.30 (<0.001)	−0.40 (<0.001)	−0.23 (0.002)	1

### Factors influencing PTSD

Multiple regression analyses were conducted to identify factors that are independently related to PTSD. Variance inflation factors (VIFs) and tolerance values were confirmed for multicollinearity. There were no multicollinearity issues. The VIFs were 1.241–5.152 (and therefore, smaller than the reference value of 10), and the range of tolerance values was 0.194–0.806 (and thus, larger than 0.1 but did not exceed 10). The independence of the residuals was checked using the Durbin-Watson statistic, which was 2.082, and there was no problem with autocorrelation. Cook's distance for outliers was 0.061 or smaller, and thus, were all smaller than the reference value of 1.0, and the assumptions for the multiple regression analyses were satisfied.

Table 5 shows the results of the regression analysis. The model was found to be significant (F = 6.41, *p* ≤ 0.001), and the adjusted coefficient of determination (Adj R^2^), which indicates the explanatory power of the model, was 0.287. Significant factors related to PTSD included having a very healthy status (B = −0.44, *p* = 0.013), CIT (B = 0.18, *p* ≤ 0.001), social support (B = −0.38, *p* ≤ 0.001), and resilience (B = −0.18, *p* = 0.044). These factors explained 28.7% of the variance in PTSD.

**Table 5:** Factors influencing post–traumatic stress disorder of police officers.

**Variables**	**Category**	**B**	**SE**	**β**	**t**	***p***
(Constant)		2.56	0.38		6.83	<0.001
Age	20–29	0				
	30–39	−0.08	0.14	−0.06	−0.58	0.566
	40–49	0.02	0.16	0.02	0.15	0.881
	50 and over	0.13	0.23	0.08	0.56	0.576
Field experience	< 5 years	0				
	5 < 10 years	0.11	0.11	0.08	1.05	0.294
	10 < 15 years	0.20	0.16	0.09	1.20	0.232
	15 < 20 years	0.26	0.21	0.12	1.25	0.213
	20 < 25 years	−0.22	0.25	−0.09	−0.88	0.380
	Over 25 years	−0.00	0.27	0.00	−0.01	0.996
Subjective health status	Having severe or some disease	0				
	Normal	−0.25	0.16	−0.16	−1.58	0.116
	Generally healthy	−0.24	0.14	−0.18	−1.67	0.096
	Very healthy	−0.44	0.18	−0.25	−2.52	0.013
Critical incident trauma	(Score)	0.18	0.04	0.29	4.15	<0.001
Social support	(Score)	−0.38	0.10	−0.28	−3.68	<0.001
Resilience	(Score)	−0.18	0.09	−0.15	−2.03	0.044
F(*p)*	6.41 (<0.001)					
Adjusted R^2^	0.287					

### Content analysis of semi-structured interviews

For the semi-structured interviews with stakeholders, all stakeholders responded that police officials suffer psychological difficulties after traumatic experiences (Table 6). The limitations of overcoming traumatic experiences were classified into five obstructive factors: social prejudice as a police official, fear of stigma and medical history of PTSD treatment, difficulty in receiving social support, fear and doubts about self-disclosure, and closed working environments. Coping strategies with traumatic experiences were divided into five categories, namely, individual response (failure), using external support, conscious separation, emotional purification, and response at the department level. The issues of anonymity, accessibility, and target selection should be considered when operating counseling programs for PTSD police officials.

**Table 6:** Content analysis of semi–structured interviews with stakeholders.

**Themes**	**Sub–themes**	**Statements (*N*)**	***N* (%)**
Psychological difficulties after traumatic experiences	Post–traumatic sequelae	After handling the decaying body, I was afraid that the virus on my body would affect the baby in the family (1).	7 (6.5)
		The memories of the events of death decades ago are vivid and cannot be easily erased (5).	
		I seem to suffer the most from insomnia and digestive problems due to stress (1).	
	Difficulty with empathy after traumatic experiences	When I found the dead body of the person who had jumped into the river, I saw the crying parents of the dead person, and as a father of two, it was hard for me to remember that scene over and over again (1).	8 (7.5)
		The crime scene corpse image was so similar to my mother's outfit that it was not easily forgotten (2).	
		I struggled for months, unable to sleep at night when a close colleague committed suicide (5).	
Limitations of overcoming traumatic experiences	Social prejudice as a police official	Many people personally give me advice that I can overcome PTSD or get better with time. If I can't overcome the traumatic incident, I feel burdened by the view that I have no qualifications as a police officer, so I often go through it on my own (4).	11 (10.3)
		There was no special way, so I had to just put up with the scary feeling (2).	
		It is difficult for me to control my emotions by myself because I am a police officer at the scene of trauma (2).	
		Even though police officers suffer from PTSD, they are often not honest with their surveys because of the perception that they have to endure professionally (3).	
	Fear of having stigma and a medical history of PTSD treatment	I was mentally exhausted, so I tried to see a psychiatrist, but I didn't get treatment because it was recorded (4).	6 (5.6)
		People with PTSD tend to use psychiatrists in other regions, or because of the prejudice that only psychiatric patients go to psychiatrists (2).	
	Due to the specificity of their work, it is difficult to receive social support	Police officers have no choice but to hide their PTSD because they can be disadvantaged even in peer evaluations based on the fact that they have experienced PTSD (3).	11 (10.3)
		My father was also a police officer but PTSD is not a positive topic; therefore, it is difficult to share it with my family (2).	
		My colleagues around me are having the same problems and are suffering mentally, so it is difficult to speak up on my own (6).	
	Fears and doubts about self–disclosure	While answering the questionnaire, I checked some items lower than the actual value because I thought, 'If I answer like this, will I be exposed?'(7)	8 (7.5)
	Closed working environments	It was difficult when a colleague committed suicide, but there was no comfort or support system around me, and I was rather inspected (1)	
The coping strategies with traumatic experiences	Individual response: failure	I can't seem to find a solution myself (3).	4 (3.7)
		I just want time to pass, and there is no other way to deal with it (1).	
	Use of external support	It seems that each of them solved it in their own way, such as by drinking, exercising, or going to church (2). I received a lot of support from my family, which worked positively and helped me overcome difficulties (1).	3 (2.8)
	Consciously separate work and personal problems	I'm trying to separate myself from my job (3).	3 (2.8)
	Individual response Capability: Emotional Purification (catharsis)	I tend to purify the whole process of the investigation and tell the truth to my family (1).	2 (1.9)
		In fact, I think the conversation is the most important thing (1).	
	How to deal with the department level	If the job is difficult, the department is transferred to a general office position (1). People who feel extreme stress cope with it by taking a leave of absence of 1 to 2 years (1).	2 (1.9)
Police Counseling Program Status	Absence of counseling programs	Although I need after–service mentally, I am currently only responding to myself, and there is no education related to PTSD (3).	3 (2.8)
	Low utility and problems of programs due to unguaranteed anonymity	When a police officer applies for a healing camp, he or she finds out who applied (3).	9 (8.4)
		Healing camps do not have many opportunities to the extent that only 2–3 people out of 100 can attend, and it seems that there are many cases wherein support is provided for relaxation rather than counseling (3).	
		To attend that kind of program, someone else has to replace the work for me (3).	
Opinions on PTSD–related program	The idea of program operations	It would be nice to have a counseling vehicle that regularly visits police agencies, like a blood donation vehicle (3).	15 (14.0)
		I wish there was a program for anonymous counseling inside the police station (5).	
		I think it would be good to make the PTSD questionnaire compulsory at the time of health check–up (2).	
		Sometimes, I wish that counseling and treatment were possible even through coercion (4).	
		Leader cooperation is an important key factor for the program to spread through the organization (1).	
	The importance of anonymity and accessibility	If anonymity and accessibility are guaranteed, it seems to be fully usable (7).	13 (12.1)
		Anonymity must be ensured so that one–on–one consultations or identity cannot be disclosed (5).	
		In the case of the current counseling program, accessibility is poor, which should be taken into consideration (1).	
	The problem of selecting candidates	It seems difficult to find subjects with high PTSD. Even if there was a suicide incident, it is often not expressed normally (2).	2 (1.9)
5	17	Total	107 (100)

## Discussion

We aimed to investigate the factors affecting PTSD among Korean police officers.

In analyzing the differences in CIT, social support, resilience, and PTSD according to participants' general characteristics, we found that male police officers have higher CIT scores. This is confirmed by the fact that male officers have more field experience than female officers. Therefore, male police officers are more likely to encounter traumatic incidents than female police officers. We found that participants aged between 20 and 29 years had higher social support scores than those aged 50 years and over. Participants aged 50 years and over had higher PTSD scores than those aged 20–29 years. These findings are consistent with previous findings (22, 23). Therefore, it is necessary to implement PTSD intervention programs for the group aged 50 years and over with low social support and high PTSD levels. We found that participants with university education had lower resilience than those with postgraduate education or higher. However, a previous study found no differences between educational level and resilience (24). Therefore, a follow-up study is needed.

Regarding rank, we also found that lower-ranked policemen had higher social support than senior inspectors. Although this result cannot be easily interpreted because there are no existing studies on this topic, Luceno-Moreno et al. (25) revealed that lower-ranked policemen had higher organizational support than higher-ranked policemen. In addition, this result is consistent with social support results based on age. As for field experience, participants with 15 to < 20 years of field experience had higher PTSD scores than those with < 5 years of experience. This result is consistent with that of Kim (26), indicating that long exposure to trauma is associated with higher PTSD scores; therefore, these participants should be selected as a high-risk group.

Second, the negative correlations between social support and PTSD as well as resilience and PTSD showed that higher social support and resilience had positive effects on PTSD, which is consistent with the results of previous studies (27, 28). Additionally, the positive correlation between social support and resilience was also reported by Mesidor and Sly (28). A positive correlation between CIT and resilience was confirmed. This is in line with the findings of Vincent (29), who showed that providing police officers with greater resilience after serious critical incidents can prevent serious mental health problems. The positive correlation between CIT and PTSD is the same as the results of Ntatamala and Adams (30) and also supports the argument that the more critical incidents police officers are exposed to, the more severe their PTSD (31).

The factors that influence PTSD include health status (very healthy), CIT, social support, and resilience. A subjective health status (very healthy) results in a lower degree of PTSD, as confirmed by previous studies (32, 33). Thormar et al. (33) found high levels of PTSD symptoms and subjective health complaints at 18 months post-disaster. In other words, PTSD also forms an area of mental health, and perceived subjective health status (very healthy) is related to lower PTSD symptoms. This study revealed that CIT influences PTSD, which confirms the findings of Bogaerts et al. (34) study, in which security workers with critical incidents suffered more PTSD symptoms and were significantly more unstable than those with no or indirect critical incidents. Similarly, Ménard and Arter (4) reported that critical incidents were positively associated with PTSD symptoms among police officers. Therefore, preventive programs related to PTSD are needed for police officers who are frequently exposed to CIT. In this study, social support and resilience were found to lower PTSD symptoms. Studies have also found that social support (10, 28) and resilience (10, 17, 28) are protective factors against PTSD. Therefore, future programs should include these components to overcome and prevent PTSD.

The results of the semi-structured interviews with stakeholders revealed that police officers are unable to easily express their mental stress due to social prejudice. Despite experiencing PTSD, it is difficult for police officers to receive social support from colleagues and families. In addition, they cannot receive appropriate psychiatric treatment because of their personal evaluations as police officers. Therefore, PTSD programs with enhanced anonymity and accessibility are necessary.

To prevent prevalence of PTSD in Korean police officers, even if they experience frequent traumatic events, it is necessary for police organizations to make efforts to strengthen their social support and resilience. In particular, there is an urgent need to prepare practical and policy measures to provide direct assistance to police officers in their work performance and to provide more active interventions for police officers who experience trauma.

## Conclusion

The critical results obtained in this study are as follows:

First, subjective health status (very healthy), CIT, social support, and resilience appeared to have strong effects on PTSD.

Second, it is important to identify the difficulties faced after post-traumatic experiences, the uniqueness of the police organization, coping strategies, and the current status of support programs for police officers in South Korea.

However, this study has some limitations that must be addressed in future studies. First, the participants were recruited through convenience sampling. Nonetheless, the results are meaningful. The vulnerability of quantitative research results was strengthened through the semi-structured interviews. In particular, mixed-methods research should be applied because of the closeness and specificity of the police organization.

Third, our findings are subject to the limitations inherent in the cross-sectional data. Therefore, in future studies, causal relationships among variables should be analyzed using longitudinal study designs.

## Data availability statement

The raw data supporting the conclusions of this article will be made available by the authors, without undue reservation.

## Ethics statement

The studies involving human participants were reviewed and approved by the Ethics Committee of Daegu University. Written informed consent to participate in this study was provided by the participants' legal guardian/next of kin. The patients/participants provided their written informed consent to participate in this study.

## Author contributions

H-KO and MK designed the study. H-KO, MK, and CJ conducted the study and collected the data. H-KO coordinated and supervised the data collection, analyzed the data, and drafted the manuscript. H-KO and CJ reviewed and revised the manuscript. All authors read and approved the final manuscript.

## References

[1] Maguen S Metzler TJ McCaslin SE Inslicht SS Henn-Haase C Neylan TC. Routine work environment stress and PTSD symptoms in police officers. J Nerv Ment Dis. (2009) 197:754–60. 10.1097/NMD.0b013e3181b975f8 19829204 PMC3974929

[2] McCanlies EC Mnatsakanova A Andrew ME Burchfiel CM Violanti JM. Positive psychological factors are associated with lower PTSD symptoms among police officers: post Hurricane Katrina. Stress Health. (2014) 30:405–15. 10.1002/smi.2615 25476965 PMC4676265

[3] Clark R Distelrath C Vaquera G Winterich D Dezolt E. Critical-incident trauma and crime scene investigation: a review of police organizational challenges and interventions. J Foren Identification. (2015) 65:929–51.

[4] Ménard KS Arter ML. Police officer alcohol use and trauma symptoms: Associations with critical incidents, coping, and social stressors. Int J Stress Manag. (2013) 20:37–56. 10.1037/a0031434

[5] Korea Government National Action Plan for Suicide Prevention. Available online at: https://www.opm.go.kr/flexer/view.do?ftype=hwp&attachNo=94247 (accessed August 10, 2022).

[6] Maeil Business Newspaper. 21 Police Officers Committed Suicide this Year and one Counselor is Consulting 427 Police Officers. Seoul: Maeil Business Newspaper.

[7] Cohen S Hoberman HM. Positive events and social supports as buffers of life change stress. J Appl Soc Psychol. (1983) 13:99–125. 10.1111/j.1559-1816.1983.tb02325.x

[8] Blais RK Tirone V Orlowska D Lofgreen A Klassen B Held P. Self-reported PTSD symptoms and social support in U.S. military service members and veterans: a meta-analysis. Europ J Psychotraumatol. (2021) 12:1851078. 10.1080/20008198.2020.1851078 34992740 PMC8725779

[9] Kwak D-G Kim D-W Kim MG. A study on the post-traumatic stress disorder in police office. Korean Assoc Criminal Psychol. (2011) 7:23–46.

[10] McCanlies EC Gu JK Andrew ME Burchfiel CM Violanti JM. Resilience mediates the relationship between social support and post-traumatic stress symptoms in police officers. J Emerg Manag. (2017) 15:107–116. 10.5055/jem.2017.0319 28820229 PMC6358175

[11] Olff M Koch SBJ Nawijn L Frijling JL Van Zuiden M Veltman DJ. Social support, oxytocin, and PTSD. Eur J Psychotraumatol. (2014) 5:26513. 10.3402/ejpt.v5.26513 25511718 PMC4265184

[12] Horn SR Charney DS Feder A. Understanding resilience: new approaches for preventing and treating PTSD. Exp Neurol. (2016) 284:119–32. 10.1016/j.expneurol.2016.07.002 27417856

[13] Southwick SM Bonanno GA Masten AS Panter-Brick C Yehuda R. Resilience definitions, theory, and challenges: interdisciplinary perspectives. Eur J Psychotraumatol. (2014) 5:25338. 10.3402/ejpt.v5.25338 25317257 PMC4185134

[14] Streb M Häller P Michael TPTSD. in paramedics: resilience and sense of coherence. Behav Cogn Psychother. (2014) 42:452–63. 10.1017/S1352465813000337 23714206

[15] Kim BH Park JP. A study on the survey of post-traumatic stress disorder of Korea police officers and systematic counter-measures. Korean J Public Safety Criminal Justice. (2016) 64:109–38. 10.21181/KJPC.2016.25.3.109

[16] Kim BH. Effect of police office's trauma aase experience on post-traumatic stress disorder [the analysis of casual effects among traumatic events, PTSD in the police officers–focused on the police officers in Busan Metropolitan. Korean J Public Safety Criminal Justice. (2016) 25:119–44. 10.21181/KJPC.2016.25.4.119

[17] Kim SH. Effects of resilience on the post-traumatic stress disorder of police officers. Korean J Public Safety Criminal Justice. (2018) 27:69–90. 10.21181/KJPC.27.2.69

[18] National Center for PTSD (2018). Life Events Checklist for DSM-5. Available online at: https://www.ptsd.va.gov/professional/assessment/te-measures/life_events_checklist.asp (accessed August 10, 2022).

[19] Kim DH Lee HK Kim JW Lee K. Reliability and validity of the Korean version of interpersonal support evaluation and List-12 (ISEL-12). Korean Neuropsychiatr Assoc. (2012) 51:416–21. 10.4306/jknpa.2012.51.6.416

[20] Liebenberg L Moore JCA. social ecological measure of resilience for adults: The RRC-ARM. Soc Indicators Res. (2018) 136:1–19. 10.1007/s11205-016-1523-y

[21] Kim J Chung H Choi J So H Kang S Kim DS. Psychometric properties of the korean version of the PTSD Checklist-5 in Elderly Korean Veterans of the Vietnam War. J Anxiety Mood. (2017) 13:123–31. 10.24986/anxmod.2017.13.2.123

[22] Lee JH Kim I Won J-U Roh J. Post-traumatic stress disorder and occupational characteristics of police officers in Republic of Korea: a cross-sectional study. BMJ Open. (2016) 6:e009937. 10.1136/bmjopen-2015-009937 26951212 PMC4785329

[23] Lee S Jeong S Choi Y. Factors affecting social support and resilience on police officer. Int J Curr Res Rev. (2021) 13:4–10. 10.31782/IJCRR.2021.13528

[24] Talavera-Velasco B Luceño-Moreno L García-Albuerne Y Martín-García J. Perception of health, resilience, and engagement in spanish police officers during the COVID-19 pandemic. Psicothema. (2021) 33:556–63. 34668469 10.7334/psicothema2021.153

[25] Luceno-Moreno L Garcia-Albuerne Y Talavera-Velasco B Martin-Garcia J. Stress in Spanish police force depending on occupational rank, sex, age and work-shift. Psicothema. (2016) 28:389–93. 10.7334/psicothema2015.310 27776606

[26] Kim JKA. Study on the post traumatic stress disorder of police officer. Korean Assoc Police Sci Rev. (2012) 36:31.

[27] Lee JK Choi HG Kim JY Nam J Kang HT Koh SB. Self-resilience as a protective factor against development of post-traumatic stress disorder symptoms in police officers. Ann Occup Environ Med. (2016) 28:58. 10.1186/s40557-016-0145-9 27777782 PMC5067890

[28] Mesidor JK Sly KF. Religious coping, general coping strategies, perceived social support, PTSD symptoms, resilience, and posttraumatic growth among survivors of the 2010 earthquake in Haiti. Ment Health Relig Cult. (2019) 22:130–43. 10.1080/13674676.2019.1580254

[29] Vincent M. The Relationship Between Programming After Critical Incidents, Shootings, and Resilience in Police. ProQuest Dissertations Publishing

[30] Ntatamala I Adams S. The correlates of post-traumatic stress disorder in ambulance personnel and barriers faced in accessing care for work-related stress. Int J Environ Res Public Health. (2022) 19:2046. 10.3390/ijerph19042046 35206234 PMC8871647

[31] Clair ME. The Relationship Between Critical Incidents, Hostility and PTSD Symptoms in Police Officers. ProQuest Dissertations Publishing.

[32] Kim SY. An analysis of factors influencing post-traumatic stress disorder (PTSD) - difference by disaster information. Crisisonomy. (2020) 16:65–78. 10.14251/crisisonomy.2020.16.5.65

[33] Thormar SB Gersons BPR Juen B Djakababa MN Karlsson T Olff M. The impact of disaster work on community volunteers: the role of peri-traumatic distress, level of personal affectedness, sleep quality and resource loss, on post-traumatic stress disorder symptoms and subjective health. J Anxiety Dis. (2014) 28:971–77. 10.1016/j.janxdis.2014.10.006 25445088

[34] Bogaerts S Daalder AL Van Der Knaap LM Kunst 4M JJ M Buschman J. Critical incident, adult attachment style, and posttraumatic stress disorder: a comparison of three groups of security workers. Soc Behav Personality. (2008) 36: 1063–72. 10.2224/sbp.2008.36.8.1063

